# Dose-Response on the Chemopreventive Effects of Sarcophine-Diol on UVB-Induced Skin Tumor Development in SKH-1 Hairless Mice

**DOI:** 10.3390/md10092111

**Published:** 2012-09-24

**Authors:** Ruth F. Guillermo, Xiaoying Zhang, Radhey S. Kaushik, David Zeman, Safwat A. Ahmed, Sherief Khalifa, Hesham Fahmy, Chandradhar Dwivedi

**Affiliations:** 1 Department of Pharmaceutical Sciences, South Dakota State University, Brookings, SD 57007, USA; Email: Ruth.Guillermo@sdstate.edu (R.F.G.); Hesham.Fahmy@sdstate.edu (H.F.); 2 ACEA Bio Ltd., Hangzhou 310030, Zhejiang, China; Email: Violett.Zhang@aceabio.com.cn; 3 Department of Biology/Microbiology, South Dakota State University, Brookings, SD 57007, USA; Email: Radhey.Kaushik@sdstate.edu; 4 Department of Veterinary and Biomedical Sciences, South Dakota State University, Brookings, SD 57007, USA; Email: David.Zeman@sdstate.edu; 5 Department of Pharmacognosy, Faculty of Pharmacy, Suez Canal University, Ismailia 41522, Egypt; Email: safwat_aa@yahoo.com; 6 College of Pharmacy, Qatar University, Doha 02713, Qatar; Email: sherief@qu.edu.qa

**Keywords:** sarcophine-diol, skin cancer, chemopreventive agent, SKH-1 mice, UVB radiation

## Abstract

Sarcophine-diol (SD) is a lactone ring-opened analogue of sarcophine. It has shown chemopreventive effects on chemically-induced skin tumor development in female CD-1 mice, as well as in a UVB-induced skin tumor development model in hairless SKH-1 mice at a dose of 30 μg SD applied topically and 180 mJ/cm^2^ UVB. The objective of this study was to determine the dose-response on the chemopreventive effects of SD on SKH-1 hairless mice when exposed to a UVB radiation dose of 30 mJ/cm^2^. This UVB dose better represents chronic human skin exposure to sunlight leading to skin cancer than previous studies applying much higher UVB doses. Carcinogenesis was initiated and promoted by UVB radiation. Female hairless SKH-1 mice were divided into five groups. The control group was topically treated with 200 μL of acetone (vehicle), and the SD treatment groups were topically treated with SD (30 μg, 45 μg, and 60 μg dissolved in 200 μL of acetone) 1 h before UVB radiation (30 mJ/cm^2^). The last group of animals received 60 μg SD/200 μL acetone without UVB exposure. These treatments were continued for 27 weeks. Tumor multiplicity and tumor volumes were recorded on a weekly basis for 27 weeks. Weight gain and any signs of toxicity were also closely monitored. Histological characteristics and the proliferating cell nuclear antigen (PCNA) were evaluated in the mice skin collected at the end of the experiment. The dose-response study proved a modest increase in chemopreventive effects with the increase in SD dose. SD reduced the number of cells positively stained with PCNA proliferation marker in mice skin. The study also showed that SD application without UVB exposure has no effect on the structure of skin. The results from this study suggest that broader range doses of SD are necessary to improve the chemopreventive effects.

## 1. Introduction

Currently, between two and three million non-melanoma skin cancers occur globally each year. One in every three cancers diagnosed is a skin cancer and, according to Skin Cancer Foundation Statistics, one in every five Americans will develop skin cancer in their lifetime [1]. American Cancer Society estimates indicated 12,190 deaths from skin cancer in 2012 [2]. The main cause for skin cancer is the excessive exposure to UV radiation from sunlight and/or sunlamps used in indoor tanning [3]. UVB is an important component of solar radiation that acts as a complete carcinogen by initiating and promoting skin cancer [4,5]. UVB is frequently used to induce photocarcinogenesis in animals. UVB radiation induces characteristic DNA damage on skin cells, immunosuppression, and modulation of various signal transduction pathways which can lead to cell proliferation, transformation, and cell death [6,7,8]. 

**Figure 1:**
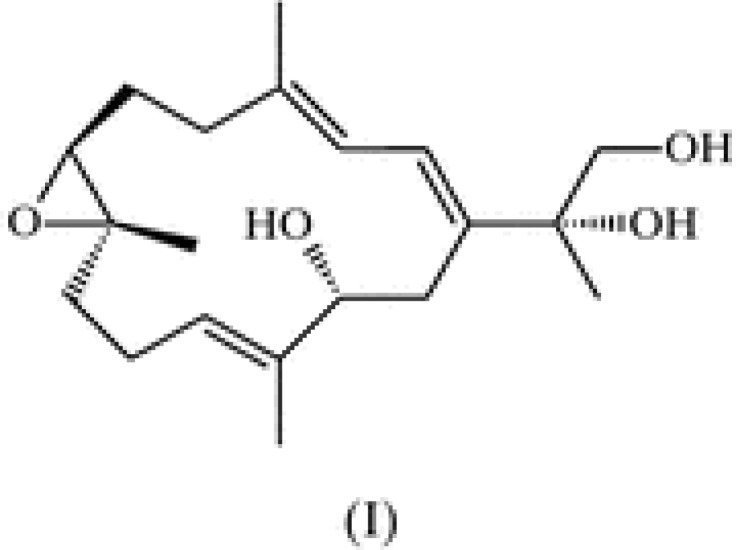
Structure of sarcophine-diol (SD).

Epidemiological data suggest that sunscreens alone are not sufficient for preventing skin cancer due to the need of frequent reapplication and the variability among commercially available sunscreens. Thus, there is a need for more effective ways to prevent skin cancer [9,10]. Recently, marine organism compounds have gained attention for their potential as chemopreventive agents due to their considerable biodiversity [11,12,13]. One of those marine compounds is sarcophine which is extracted from the soft coral *Sarcophyton glaucum*. One of the semisynthetic derivatives of sarcophine is sarcophine-diol (SD, Figure 1) [14,15,16]. Our laboratory has reported its chemopreventive effects against chemically-induced skin carcinogenesis in CD-1 mice [17]. Also, SD at 30 μg/dose in a UVB (180 mJ/cm^2^) induced skin carcinogenesis in a SKH-1 hairless mice model significantly inhibited tumor multiplicity and tumor area, without signs of toxicity in the animals. In the same experiment, SD increased the activation of cleaved caspases 3 and 8 in mice skin tissue [18]. Mechanistic studies performed in our laboratory showed that SD decreased cell viability and cell proliferation of the A431 squamous carcinoma cell line in a dose and time-dependent manner and caused apoptosis and DNA fragmentation [19]. Recent reports showed that SD has antineoplastic effects *in vitro* in the mouse melanoma cell line B_16_F_10_[20]. 

Proliferating cell nuclear antigen (PCNA) is an auxiliary protein necessary for DNA synthesis [21] and DNA repair [22]. PCNA is used as a marker of cell proliferation in tissues to assess the efficiency of chemopreventive drugs in cancer research [23,24]. An abnormally high abundance of PCNA-positive cells has been reported in squamous cell carcinoma as compared to other keratinocytic neoplasms [25] and when compared to other non-malignant skin diseases [26]. Immunohistochemical studies also have shown increased nuclear PCNA staining in clinically and histologically aggressive basal cell carcinoma (BCC) [27]. We evaluated PCNA staining in skin sections as a marker of cell proliferation in mice skin.

The objective of this study was to determine the chemopreventive effects of SD when applied at different doses (30, 45, 60 μg) on a chronic and low dose (30 mJ/cm^2^) UVB-induced skin tumor development model in female SKH-1 hairless mice, as this is an experimental model more relevant to human skin cancer development than other investigations using higher UVB doses [28]. 

## 2. Results and Discussion

### 2.1. SD Treatment Did Not Affect Body Weight of SKH-1 Hairless Mice

There was no significant difference in weight gain among various groups treated with sarcophine-diol or acetone (data not shown). 

### 2.2. SD Treatment Alone Did Not Affect the Skin of SKH-1 Hairless Mice

The group of animals being treated with 60 μg SD, but no UVB showed no apparent toxicity and no change on histological examination as compared with untreated control mice skin (pictures not shown). In the skin of matching animals without SD treatment and without UVB exposure, it was observed that the epidermis is four cell layers thick. The epidermis is covered by a thin layer of normal basket-weave keratin. Hair follicles appear poorly developed; hair shafts are rare. There are small sebaceous glands near the infundibulum of most follicles. Sweat glands are dilated and prominent throughout. The collagen in the dermis is compact, dense and brightly eosinophilic. A few small clusters of mast cells are in the dermis. This skin is very comparable to mice in the study that did not receive UVB treatments but were applied with SD 60 μg/dose in the same fashion as the other treatment groups. This would suggest that SD alone had no adverse effect on skin morphology. 

### 2.3. SD Treatment Did Not Significantly Inhibit the Incidence of Skin Tumors in SKH-1 Hairless Mice

**Figure 2:**
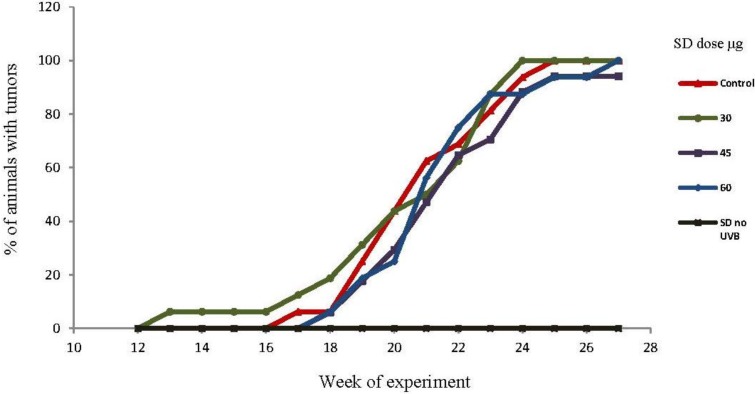
Effects of SD treatment on tumor incidence in SKH-1 mice. SD pre-treatment did not affect the incidence of tumors (*n*=20 per group). By the 13th week, one animal from the 30 μg/dose group developed one tumor. During the week 17th, animals from the control group started developing tumors, while tumors started appearing by the 18th week in the animals treated with 45 and 60 μg/dose. By the 25th week, the incidence for all groups ranged from 95% to 100%. The group treated with 60 μg/dose of SD and no UVB exposure, did not develop tumors at any time during the experiment.

Tumor incidence is defined as the percentage of mice bearing at least one tumor on the treated area. The effects of SD treatment on the tumor incidence are shown in Figure 2. By the 18th week at least one animal in all groups presented at least one tumor, except the group not exposed to UVB. By the end of the experiment, at week 27, all UVB-exposed groups had a tumor incidence rate between 95%–100%. Except for the one animal that developed one tumor at week 13th into the protocol, the chronology of the tumorigenesis is consistent with previous reports from our laboratory, using a similar UVB dose/protocol in SKH-1 mice [29,30]. The reasons why that one animal in the 30 μg/dose group developed one tumor earlier in the experiment can be explained by the fact that they are outbred animals and may have different immune response when insulted by the UVB radiation. As with people, some individuals may be more susceptible to the UVB skin damage, having less repair mechanism, less natural apoptotic response or other factors that we did not explore in this particular experiment. Results showed that SD pre-treatment in concentrations of 30, 45, 60 μg/dose did not have significant (*P* < 0.05) effects on the incidence of tumors throughout the experiment. 

### 2.4. SD Treatment Inhibited Tumor Multiplicity in SKH-1 Mice

**Figure 3:**
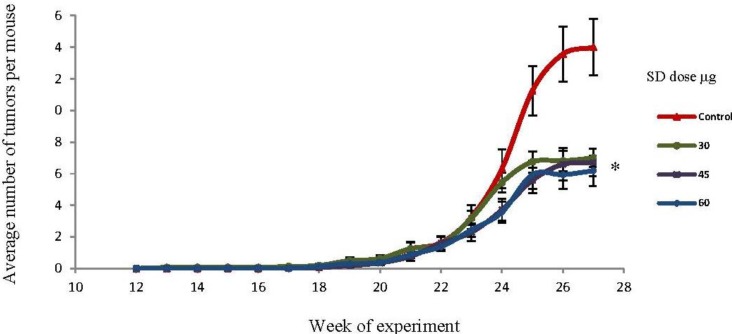
Effects of SD treatment on tumor multiplicity in SKH-1 mice. SD pretreatment significantly (*P* <0.05) decreased tumor multiplicity in all groups by the end of the experiment. Each point represents mean number of tumors per mice ± SEM derived from 20 mice in each group. * Significantly different.

The effects of SD in tumor multiplicity (average number of tumors per mouse) are presented in Figure 3. At the 27th week, the mean number of tumors per mouse was 14, 7, 6.7, and 6.2 for the control, 30 μg, 45 μg, and 60 μg SD groups respectively. This accounts for a 50%, 52.1%, and 55.8% inhibition in tumor multiplicity for 30, 45, and 60 μg/dose respectively. This model showed even stronger SD effects than a previous study, where we found a 36% inhibition rate in tumor multiplicity with SD (30 μg/dose) as compared to acetone (UVB dose: 180 mJ/cm^2^) [18]. Overall, SD pre-treatment resulted in a significant (*P* < 0.05) reduction in tumor multiplicity in all groups. However, there were not significant differences in tumor multiplicity among the different treatment groups (SD 30, 45, 60 μg).

### 2.5. SD Treatment Inhibited Tumor Volume in SKH-1 Mice

**Figure 4:**
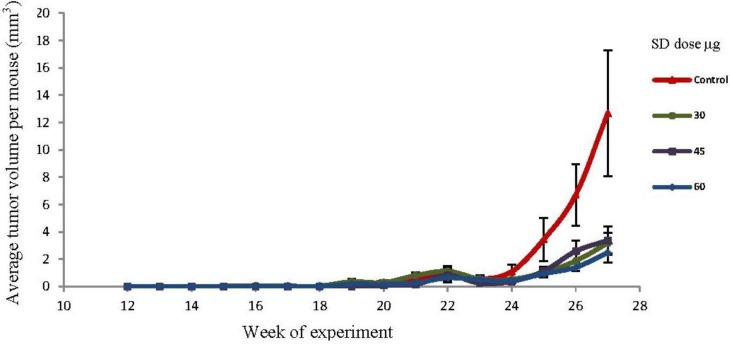
Effects of SD treatment on tumor volume in SKH-1 mice. Each point represents the average tumor volume (mm^3^) per mouse ± SEM. Tumors were measured as described in experimental section. Tumor volumes were similar in all three SD treatments groups (*n* =20).

By the end of the experiment (27th week) there was a reduction in the average tumor volume per mouse (mm^3^) of 74.9%, 73.3% and 80.5% respectively for the 30 μg, 45 μg and 60 μg SD treated groups as compared to control. However, these reductions in tumor volumes were not statistically significant because of the high standard deviation for tumor volume in the control group. The effects of SD on tumor volume are presented in Figure 4. Each point represents the average tumor volume (mm^3^) per mouse ± SEM. The length, width, and height of each tumor were measured with Vernier caliper and the formula 4/3 πr^2^ was applied to calculate the tumor volumes.

### 2.6. SD Inhibits UVB-Induced PCNA Positive Cells on Mice Skin

**Figure 5:**
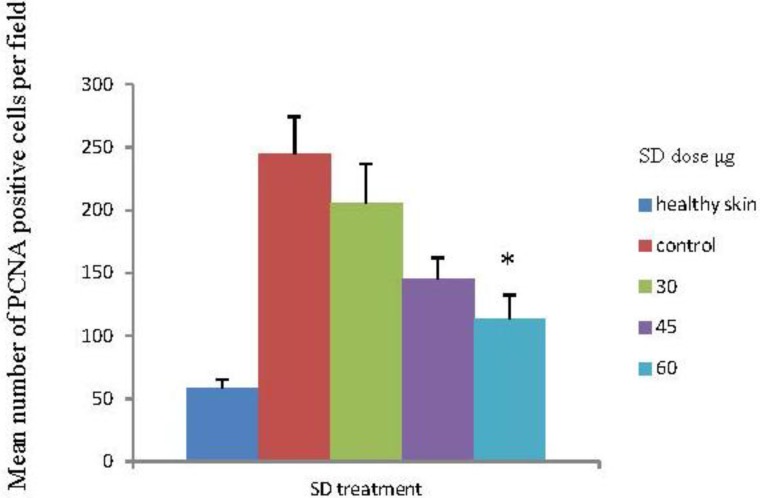
Effects of SD treatment on proliferating cell nuclear antigen (PCNA) positive cells in UVB exposed SKH-1 mice skin. Expression of PCNA in skin tumors was evaluated by immunohistochemistry. Skin samples from four randomly selected mice per group were stained for PCNA. The PCNA positive cells were counted in four fields per each skin sample section. Each bar represents the mean number of PCNA positive cells ± SEM from the sixteen measurements per group.

**Figure 6:**
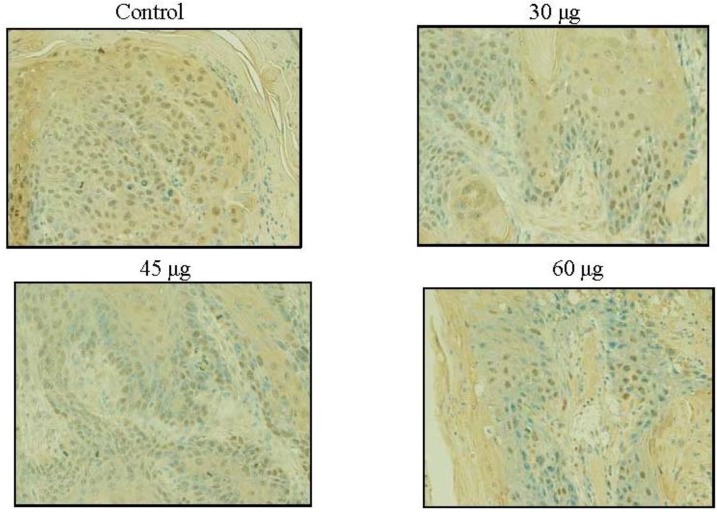
Effects of SD treatment on PCNA positive cells in SKH-1 mice. SD inhibited UVB-induced PCNA expression in the mice skin. All skin samples were collected at the end of the experiment and skins from four random animals per group were used for preparing the sections and four panels from each slide were analyzed. Photographs were taken at 200× and the numbers of positively stained cells were counted. The PCNA staining appears dark brown.

Skin exposure to UVB radiation enhances the proliferation potential of skin cells, reflected in an increase on PCNA positive cells in the epidermis and dermis [31]. Besides causing a significant reduction in the average number of tumors per mouse (Figure 3), SD treatment under all three doses resulted in a reduction in the number of PCNA-positive cells in the skin sections as compared to the control group (acetone treated), seen in Figure 5. Reductions in the number of PCNA positive cells were 16%, 41% and 53.6% for the 30, 45 and 60 μg SD treated groups, respectively. Skin sections from the 60 μg SD group presented a significantly lower number of PCNA positive cells (*P* < 0.05) compared to the control group (UVB exposed and acetone treated), seen in Figure 6. In these experiments all skin samples for PCNA staining were collected at the end of the experiment. For a clear understanding of SD chemopreventive effects it would be necessary to assess its effects at earlier points in the experiment as well. In addition to PCNA, there are several early molecular biomarkers such as thymine dimers, apoptosis, cyclin D1, p53 and p21 that should be target for evaluation in future studies assessing the skin cancer chemopreventive effects of SD. 

### 2.7. UVB Radiation Induced Carcinoma *in Situ* and Squamous Cell Carcinoma in SD Treated and Control Groups

The histopathological evaluation of the tumors was performed by a board certified veterinary pathologist; Diplomate, ACVP (DZ) at the end of the 27th week; results showed that both control and SD treated groups developed carcinomas in situ and squamous cell carcinomas in the skin (pictures not shown). The squamous cell carcinomas (SCC) on microscopic examination reveal proliferative neoplastic lesions of the epidermis which form plaques and nodules that protrude deep into the dermis. The epidermal layer is up to 10 times its normal thickness and the surface is covered by a thick layer of keratin and cellular debris. The neoplastic cells form irregular trabeculae and large cell nests. Some cell nests form squamous keratin pearls at their centers. A typical neoplastic cell is a large polyhedral cell with abundant eosinophilic cytoplasm, and a large round nucleus which is vesiculated and contains one or two large nucleoli. Foci of dyskeratosis are noted. Mitotic figures are common and occasionally bizarre. The subjacent dermis shows mild fibroplasia and some mild reactive inflammation. The lesions identified as carcinomas in situ are very similar to that described for squamous cell carcinoma above, except that the lesion is smaller and confined to the epidermis; does not cross the basal cell layer. 

Examining non-tumorous areas of the skin on mice that received UV treatment with or without SD showed UV treatment damage to the skin. This damage is characterized by epidermal hyperplasia (10 cell layers thick *versus* 4 in untreated mice). Some cellular atypia is noted, characterized by nuclear enlargement, clumped chromatin, and dyskeratosis. In the dermis, the superficial collagen is pale and smudged in appearance.

### 2.8. Discussion

Skin cancer prevalence has increased steadily in the last few years and some academics propose that non-melanoma skin cancer is an under recognized epidemic in the United States [32]. Annually, skin cancer costs an estimated $1.7 billion to treat and results in $3.8 billion in lost productivity [33]. The situation seems to be getting worse since the usage of tanning devices is increasing and people are younger when they start practicing indoor tanning, increasing their lifetime risk of getting non-melanoma skin cancer [34]. Thus, there is a strong need for effective chemopreventive agents for non-melanoma skin cancer. 

In recent years there have been efforts to find chemopreventive agents that target skin cancer. One of those agents is Sarcophytol A which is extracted from the Red Sea coral *Sarcophyton glaucum*. After showing promising effects as a chemopreventive agent in pre-clinical studies [35,36,37], Sarcophytol A entered early clinical trials as a skin cancer chemopreventive agent [38] but the trials got hampered by the lack of supply of Sarcophytol A, because it is found only in minute quantities in the coral. In response to this situation, scientists explored derivatives from Sarcophine which is a more abundant cembranolide extracted from the same coral [39]. Sarcophine is a fish toxin; it is an inhibitor of enzymes such as cholinesterase and phosphofructokinase [39,40]. Sarcophine-diol [14] is a derivative from Sarcophine.

It has been reported that sarcophine-diol (SD) inhibited the Epstein-Barr virus early antigen activation in Raji cells [14,15]. Also, in a chemically induced skin cancer model in ICR mice, SD showed inhibitory effects on incidence and multiplicity of papillomas [16]. In a report from our laboratory, SD showed chemopreventive effects in a DMBA initiated and TPA promoted model in female CD-1 mice. In the same study it was found that SD increased the expression of caspase-3 and caspase-8 and decreased cyclooxygenase-2 expressions in mice skin. SD resulted in a 95% reduction in 12-*O*-tetradecanoylphorbol-13-acetate-induced DNA synthesis [17]. Further experiments are necessary to determine whether the SD doses used in the present work in SKH-1 mice are still affecting apoptosis and COX-2 expression as it was observed in the previous study in CD-1 mice [17].

Previous mechanistic studies from our laboratory in squamous carcinoma cell line A431 showed that SD treatment at concentrations of 200 to 600 μM resulted in a concentration-dependent decrease in cell viability and cell proliferation. SD treatment induced strong apoptosis and significantly increased DNA fragmentation in A431 cells. Furthermore, SD treatment significantly increased the activity and expression of caspase-3 through activation of upstream caspase-8 in A431 cells. In the same report, SD treatment was shown to be much less cytotoxic in monkey kidney CV-1 cells. These results suggest that SD decreased cell growth and induced apoptosis through extrinsic pathway in A431 cells [19]. Our laboratory performed further *in vivo* studies on topically applied SD, on female SKH-1 mice. In that experiment, the initiation and promotion phases were induced by UVB radiation (180 mJ/cm^2^). SD was applied topically at 30 μg/100 μL of acetone 1 h prior to UVB exposure for 14 days as initiation. During the promotion phase, both control and SD treatment groups were treated twice a week for 30 weeks. At the end of the experiment tumor multiplicity in control and SD treatment groups were 25.8 and 16.5 tumors per mouse, respectively. SD induced DNA fragmentation by increasing the expressions of cleaved caspase-3 and caspase-8. The total tumor area to total back area was 18.0% and 5.0% in control and SD treatment groups, respectively. The results from that study, using a high UVB dose (180 mJ/cm^2^), indicated that SD has potential to be a potent chemopreventive agent at higher concentrations for non-melanoma skin cancer development by inducing apoptosis through apoptotic extrinsic pathway [18]. More experiments are necessary to address if the SD doses applied in the present experiments are affecting the same pathways.

Based on those previous findings, we wanted to evaluate if increasing the SD dose and changing the UVB dose to one that is more relevant to human cancer would result in better chemopreventive effects. Human behavior of getting sunlight exposure on a daily basis is resembled in the current model where animals are exposed daily to the UVB radiation. We chose to evaluate PCNA as a proliferation marker in the tissue sections; as other groups have used PCNA as a marker for the efficacy of chemopreventive agents [41,42,43]. 

We observed a modest increase in the chemopreventive effects when we increased the SD dose: 50%, 52.1%, and 55.8% inhibition in tumor multiplicity for 30, 45, and 60 μg/dose, respectively. For the tumor volume we reported an inhibition of 74.9%, 73.3% and 80.5% respectively for the 30 μg, 45 μg and 60 μg SD treated groups as compared to control. However, the reductions in tumor volumes were not statistically significant because of the high standard deviation for tumor volume in the control group. SD significantly reduced the average number of PCNA positive stained cells within the 60 μg group. There was no significant difference in the effect on tumor incidence within the three treated groups. SD at any of the studied doses had no effect on the tumor incidence. Another important observation from our study is that SD at 60 μg per application for 27 weeks was apparently innocuous to the skin and general health, since the animals exhibited no macro or microscopic difference when compared to matched mice without any treatment. This suggests safety in using SD at 60 μg, applied topically. SD did not affect the characteristics of the tumors; tumors within all four groups were mostly squamous cell carcinoma and in less number carcinoma *in situ*. SD has chemopreventive effects at very low doses: 30–60 μg/application as compared to other reported chemopreventive agents with similar effects on UVB induced skin cancer models. Such as: baicalin (1 mg/cm^2^) [41], brown algae polyphenols at 3 and 6 mg/application [43], alpha-santalol at 5 mg per application [44], (−)-epigallocatechin-3-gallate (EGCG) at (1 mg/cm^2^) [45], and silibinin at 9 mg per application [46]. 

Our results suggest that SD may need to be applied at even higher doses for achieving improved chemopreventive effects. Further studies with additional doses of SD are necessary to understand its chemopreventive potential.

## 3. Experimental Section

### 3.1. Materials and Reagents

The soft coral *Sarcophyton glaucum* was collected from several locations of the Red Sea in Egypt; *n*-hexane, ethyl acetate, and acetone were purchased from Thermo Fisher scientific (Waltham, MA, USA); the rest of the chemicals were obtained from Sigma-Aldrich (St. Louis, MO, USA). The ABC staining system and primary goat anti-PCNA antibody were obtained from Santa Cruz Biotechnology Inc., (Santa Cruz, CA, USA). Other reagents were obtained in the highest purity grade commercially available.

### 3.2. Synthesis of Sarcophine-Diol (SD)

Sarcophine was isolated from the Red Sea soft coral *Sarcophyton glaucum* by multiple extractions with petroleum ether at room temperature following the reported procedure [14]. Sarcophine-diol was synthesized as previously reported [14,15]. The structure of SD was fully characterized by spectroscopic methods (NMR) as shown in Figure 1. Purity was confirmed by HPLC.

### 3.3. Animals

Female SKH-1 mice (4–5 weeks old) were purchased from the Charles River Breeding Laboratories (Wilmington, MA). They were placed in transparent acrylic cages at 5 animals/cage, under a climate controlled environment (temperature 22 ± 1 °C, humidity 40%–60%, light 6.00–18.00 h). Mice were given water and food *ad libitum*. The experimental protocol was approved by our Institutional Animal Care and Use Committee. The animals were observed for 2 weeks before starting the experiment.

### 3.4. UVBExposureSource

The UVB light source was four FS-40-T-12-UVB sunlamps (Daavlin, Bryan, OH) emitting 80% radiation within 280–340 nm with a peak at 314 nm. The UVB exposure dose was controlled by using two Daavlin Flex Control Integrating 305 dosimeters manufactured by Daavlin Corporation (Bryan, OH, USA). UVB lumps were calibrated before and during the experiments to emit 30 mJ/cm^2^ to the dorsal section of the animals at a distance of approximately 23 cm (distance from the animal’s back and the lamps). The duration of radiation exposure was 13–16 seconds on average. 

### 3.5. UVB-Induced Skin Carcinogenesis Protocol

A total of 85 female SKH-1 hairless mice were divided into 5 groups, four groups of 20 animals and one group of five mice. The control group (*n* = 20) received 200 μL acetone topically 1 h before UVB exposure, the treatment groups had 20 mice each and received 30 μg, 45 μg, or 60 μg SD dissolved in 200 μL of acetone, while the last group (*n* = 5) received 60 μg SD/200 μL acetone and was non-irradiated. Treatments or vehicle were applied topically on the backs of the mice Monday through Friday. This experiment was carried out for 27 weeks. This protocol has been described in our previous studies [29,30].

### 3.6. Immunohistochemical Detection of PCNA-Positive Cells

For the inmunostaining of skin sections we used the goat ABC staining system (Santa Cruz Biotechnology Inc., CA, USA). We followed the vendors’ protocol. Briefly, paraffin sections of skin (5 μm thick) collected from the mice were deparaffinized in 100% xylene and re-hydrated in a descending ethanol series. Endogenous peroxidase activity was blocked with 0.5% hydrogen peroxide (Acros Organics, Geel, Belgium). Heat-induced epitope retrieval was performed in 10 mM citrate buffer at 95 °C for 7 min. The non-specific binding sites were blocked with 1.5% blocking serum in PBS. Followed by incubation with PCNA antibody (5 μg/mL, Santa Cruz Biotechnology) overnight at 4 °C in humid chamber. After washing with PBS, the sections were incubated with biotinylated secondary antibody and exposed to avidin and biotinylated horseradish peroxidase (AB reagents) and the peroxidase reaction was developed with 3,3-diaminobenzidine chromogen solution in DAB buffer substrate. Sections were counterstained with hematoxylin Mayer (Electron Microscopy Sciences, Hatfield, PA, USA), rinsed in Scott’s solution (Ricca chemicals, Arlington, TX, USA), mounted in Permount (Thermo Fisher Scientific, Waltham, MA, USA) and analyzed using a bright field microscope (AX-70, Olympus Microscopy, Hamburg, Germany). For negative control in the immunohistochemistry experiments, 1.5% blocking serum was used to replace the primary antibody.

### 3.7. Statistical Analysis

The software INSTAT (Graph Pad, San Diego, CA, USA) was used to analyze the data. Chi Square was used for analyzing the data on tumor incidence. Krushal-Wallis test (Nonparametric ANOVA) followed by Dunn’s multiple comparison test was applied to compare the tumor multiplicity, volume, weight gain and PCNA stained cells. Significance in all the cases was considered at *P* < 0.05.

## 4. Conclusions

The results from this photocarcinogenesis model suggest that SD has chemopreventive effects at 30 μg per application, increasing modestly when applied at 45 μg or 60 μg. It was observed that SD inhibited tumor multiplicity (50%–60% inhibition), tumor volume (70%–80% reduction) and the number of positive cells for the proliferation marker PCNA (16%, 40.7% and 53.6% for the 30 μg, 45 μg and 60 μg SD groups, respectively) as compared to the control (100%). Further studies using a broader range of SD doses are necessary to understand SD’s chemopreventive potential on this skin cancer model. SD did not show any evident effect on skin or animal weights when applied without concurrent UVB. Future studies on dose-response, time-response and to evaluate the mechanisms of action are needed to fully explore the skin cancer preventive effects of SD.
